# Case report: Huppke–Brendel syndrome in an adult, mistaken for and treated as Wilson disease for 25 years

**DOI:** 10.3389/fneur.2022.957794

**Published:** 2022-09-01

**Authors:** Frederik Teicher Kirk, Ditte Emilie Munk, Jakob Ek, Lisbeth Birk Møller, Mette Bendixen Thorup, Erik Hvid Danielsen, Hendrik Vilstrup, Peter Ott, Thomas Damgaard Sandahl

**Affiliations:** ^1^Department of Hepatology and Gastroenterology, Aarhus University Hospital, ERN Rare Liver, Aarhus, Denmark; ^2^Department of Genetics, Copenhagen University, Rigshospitalet, København, Denmark; ^3^Department of Radiology and MR-center, Aarhus University Hospital, Aarhus, Denmark; ^4^Department of Neurology, Aarhus University Hospital, Aarhus, Denmark

**Keywords:** Wilson disease, copper, neurology, SLC33A1, rare disease, Huppke-Brendel syndrome, case report

## Abstract

**Background:**

Huppke–Brendel (HB) syndrome is an autosomal recessive disease caused by variants in the *SLC33A1* gene. Since 2012, less than ten patients have been reported, none survived year six. With neurologic involvement and ceruloplasmin deficiency, it may mimic Wilson disease (WD).

**Objectives and methods:**

We report the first adult patient with HB.

**Results:**

The patient suffered from moderate intellectual disability, partial hearing loss, spastic ataxia, hypotonia, and unilateral tremor of parkinsonian type. At age 29, she was diagnosed with WD based on neurology, elevated 24H urinary copper, low ceruloplasmin, and pathological ^65^Cu test. Approximately 25 years later, genetic testing did not support WD or aceruloplasminemia. Full genome sequencing revealed two likely pathogenic variants in *SLC33A1* which combined with re-evaluation of neurologic symptoms and MRI suggested the diagnosis of HB.

**Conclusion:**

Adult patients with HB exist and may be confused with WD. Low ceruloplasmin and the absence of *ATP7B* variants should raise suspicion.

## Introduction

Huppke–Brendel (HB) syndrome was first described in 2012 (1). Since less than ten patients have been reported, all pediatric (2). Clinical presentation is characterized by congenital cataracts, deafness, developmental delay, and death before the age of 6. MRI shows hypomyelination, cerebral atrophy with wide subarachnoid spaces, and cerebellar hypoplasia (1, 3).

Huppke–Brendel syndrome is caused by pathogenic biallelic variants in the *SLC33A1* gene located on the long arm of chromosome 3. The protein product is acetyl-coenzyme A transporter 1 (AT-1), a highly conserved transmembrane protein located in the endoplasmic reticulum (ER) (4). AT-1 is involved in the acetylation of gangliosides and glycoproteins, by transporting acetyl-CoA from cytosol to the ER lumen (5). AT-1 dysfunction alters protein modification, delays Golgi-to-plasma protein trafficking, and increases in number of lysosomes (6). Thus AT-1 dysfunction may affect many proteins and processes due to its involvement in the secretory pathway (1, 7, 8).

One consequence of AT-1 dysfunction in HB is ceruloplasmin deficiency with low plasma copper and severe neurological phenotype. Biochemically, HB resembles other copper metabolism disorders such as Wilson disease (WD), Menkes disease, and aceruloplasminemia (1, 9).

Here, we present a Danish woman diagnosed in 1996 at age 29 with WD. In 2021, the diagnosis was revised, and HB was identified after the detection of two pathogenic variants in the *SLC33A1* gene.

## Case report

The patient was born in 1967 with developmental delays and partial hearing loss. Most of her schooling was spent in special education classes where she acquired rudimentary reading skills. In her late teenage years, she was hospitalized with anorexia nervosa and treated for depression.

### 1996–2017

In 1996, the patient, aged 29, was admitted to the hospital with unilateral Parkinsonian tremor, spastically ataxic gait disturbances, apraxia, hypotonia, and dysarthria. She was described as mentally and intellectually inferior. Dysphagia was not observed.

Clinical evaluation revealed immeasurably low serum-ceruloplasmin (S-Cp) (<0.05 g/L; normal range 0.20–0.45 g/L) and low serum-copper (S-Cu) (3, 12–23 μmol/L). 24H urinary copper excretion (24H U-Cu) was moderately elevated (6.5, 0.1–1.3 μmol/24H). Liver function tests, iron, hemoglobin, and ferritin were normal. Liver histology was normal as were copper levels (7.9 mg/kg dry weight). Slit-lamp examination revealed no Kayser–Fleischer rings. Genetic testing was unavailable. According to the Sternlieb criteria valid in 1996, typical neurological symptoms and very low ceruloplasmin were sufficient to diagnose WD, elevated 24H U-Cu supported the diagnosis (10). However, normal hepatic copper raised uncertainty and it was decided to use the ultimate diagnostic tool at time, assessment of plasma ^65^Cu after oral administration (11). As typical for WD, the test was without the secondary peak in plasma of labeled copper at 72 h seen in healthy controls. Thus, the diagnosis of WD was accepted. Revised diagnostic “Leipzig” criteria were published in 2003 (12) and did not challenge the WD diagnosis. WD was still “highly likely” with 2 points each for neurology, elevated 24H U-Cu, low ceruloplasmin, and −1 for normal liver Cu, total 5 points. The AASLD guideline in 2008 or that of EASL in 2012 (13, 14) did not change that situation.

Zinc therapy was initiated for presumed WD. Neuropsychiatric symptoms were treated with antiepileptic and antipsychotic drugs. Until 2017, she remained stable albeit psychologically vulnerable and without progression in her neurological symptoms. She lived in her own home, with her husband, but received help from healthcare workers and family to assist in daily living.

### 2017

In 2017 at age 49, the patient developed severe dysphagia over several months. She lost weight, her mobility decreased, and she lost bladder and bowel control. She was hospitalized for aspiration pneumonia and admitted to an intensive care unit. Dysphagia was thought to be a progression of WD; therefore, she was transferred to our facility for treatment optimization. Penicillamine 600 mg × 2 daily was added to the treatment regimen. The 24H U-Cu excretion was normal (0.57 μmol/24H; normal range 0.61–1.62) and increased to 3.79 μmol/24H on penicillamine. Since the symptoms could also be caused by an overdose of her antipsychotic and/or antiepileptic medication, these were reduced and discontinued, respectively. Following the medication adjustments, the dysphagia and mobility improved over 2 months.

### 2021

Lack of genetic testing and less characteristic neurology led us to revisit the diagnosis. Liver function tests and standard blood chemistry were normal. Like in 1996, both S-Cu (1.5 μmol/L; normal range: 7.9–23.6 μmol/L) and ceruloplasmin (0.03, 0.15–0.45 g/L) were low. Exchangeable copper (CuEXC) (0.81, 0.61–1.61 μmol/L) was normal and relative exchangeable copper (REC) was elevated (54%, normal range: 3–9.7%) as expected in well-treated WD, apparently confirming the diagnosis (15, 16). However, the extensive genetic analysis did not detect potentially pathogenic variants of neither the *ATP7B* gene responsible for WD nor the CP gene responsible for aceruloplasminemia.

Therefore, whole exome sequencing was performed [Twist Human Core Exome Library Kit (TWIST Bioscience, USA) and sequencing on the NovaSeq platform (Illumina, US)] to identify variants in genes associated with WD or WD-like diseases. The patient proved heterozygote for two variants: c.817_819 del, p.(Thr273del) and c.1331T>C, p.(Ile444Thr) in the *SLC33A1* gene (NM004733.4). None of these variants have been reported as pathogenic. In the gnomAD database containing more than 125,000 healthy individuals, only the c.817_819del variant is described in 3 alleles (in heterozygous form) (https://gnomad.broadinstitute.org/). The variant c.1331T>C, p.(Ile444Thr) has not been described previously. The variant, c.817_819del leads to a single amino acid deletion. Both amino acids are very evolutionary conserved and *in silico* both variants are predicted to be pathogenic (CADD scores 20.3 and 26.8, respectively). Following ACMG classification, we classify both variants as variants of uncertain significance. No clinical information is available on the patient's now deceased parents; the patient has no siblings or children.

**Figure 1:**
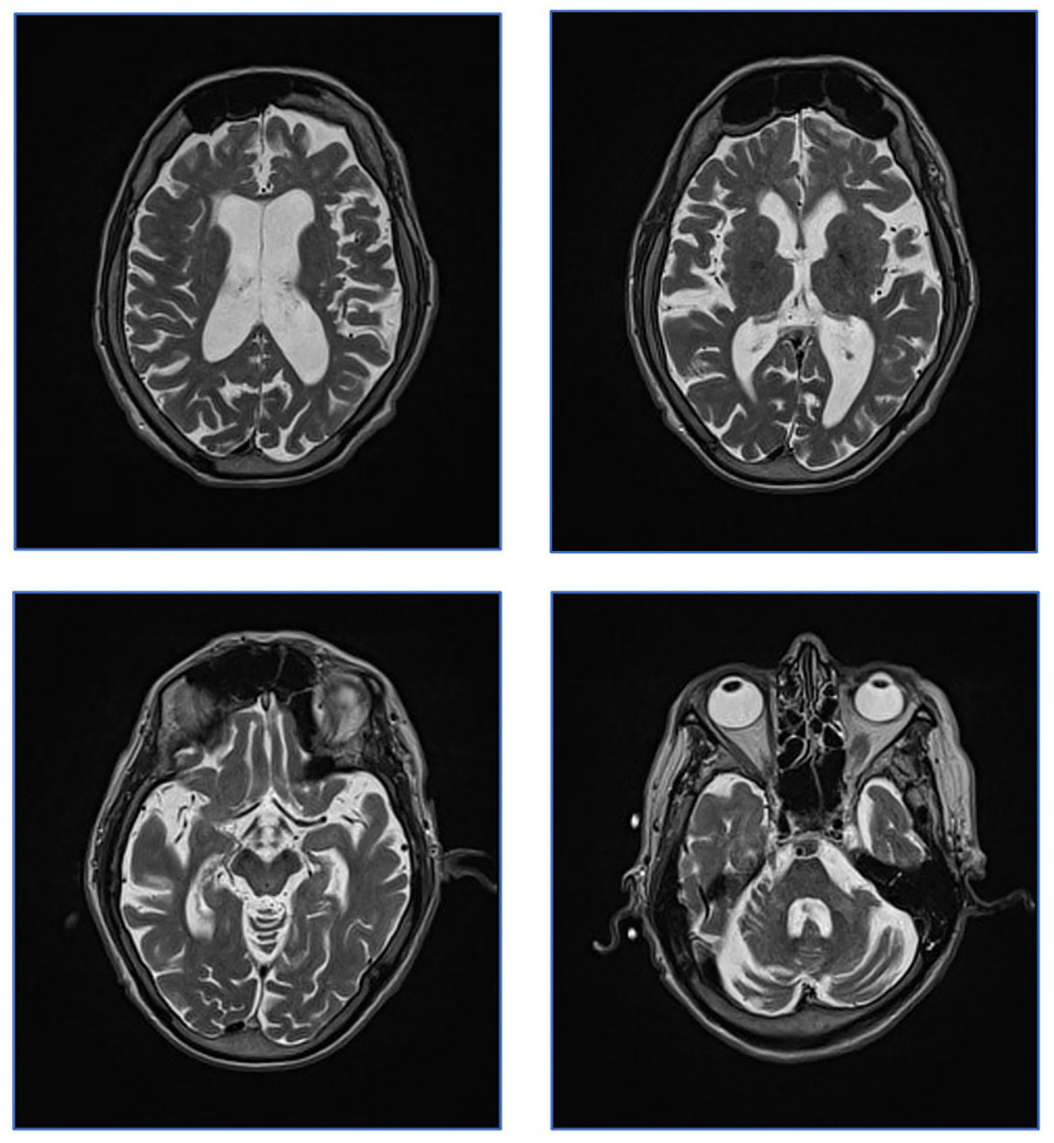
Patient MRI from 2017. Patient MRI. Axial T2 weighed images showing global atrophy and unspecific T2 hyperintensities. Characteristic WD changes to basal ganglia were not present.

Brain MRI scans from 2017 were revisited. No signal changes related to the basal ganglia and brainstem were found as would be expected in WD. Only small unspecific T2-hyperintensities were seen in the frontal lobes. Further, global atrophy including cerebellar atrophy was indicated by an ectatic ventricular system and sulcal widening (Figure 1).

Based on these findings, the most likely diagnosis was HB in a phenotypically less severe form compared to previous pediatric cases in the literature. Table 1 presents an overview of expected findings in HB, compared to other copper metabolic diseases. In support, the neurology included features such as intellectual disability and spastic ataxia that has been described in HB and has less characteristic of WD. Low S-Cp and S-Cu would be expected also in HB. So far, exchangeable copper assessment has not been examined in HB, but elevated REC could be explained by free cobber being normal and ceruloplasmin being extremely low. Without the ability to produce ceruloplasmin, the ^65^Cu test would miss the secondary 72H peak in both HB and WD. Therefore, the patient's neurological deterioration in 2017 was likely caused by large doses of neuroleptics rather than a WD manifestation.

**Table 1:** Overview of Huppke–Brendel (HB) syndrome and other copper-related diseases.

	**Wilson disease**	**HB**	**Aceruloplasminemia**	**Menkes disease**
Gene	*ATP7B*	*SLC33A1*	*CP gene*	*ATP7A*
	Autosomal	Autosomal	Autosomal	X-linked
Protein	ATP7B	AT-1	Ceruloplasmin	ATP7A
Presentation	5–40 Y typically (3-80 Y has been reported)	Pediatric 0-6 Y	20 – 60 Y	Y
		Adult?		Adult form
Neurology	Tremor, dystonia, dysarthria, Dysphagia Psychiatry Hepatic involvement.	Developmental delay, intellectual disability, hypotonia, nystagmus, bilateral congenital cataract, deafness.	Dystonia, tremor, chorea, psychiatric, midlife dementia, retinal degeneration.	Pediatric, hypopigmented brittle hair, seizures, stunted growth, failure to thrive, dystonia, and intellectual disability.
				Adult
				Distal motor neuropathy.
Typical MRI	Panda sign in midbrain, tegmentum and pons.	Global hypoplasia, hypomyelination and wide subarachnoid space.	T2* and T2 FSE hypointensity in the dentate nuclei, thalamus, and basal ganglia.	Hypomyelination, global atrophy, ventriculomegaly, and tortuosity of the cerebral vasculature.
	T2* hyperintensity of putamina and deep gray nuclei including basal ganglia.			
Ceruloplasmin	Low to normal	Absent to Low	Absent	Low
				Normal in infants
S-Cu	Low to normal	Absent to Low	Absent	Low
Hepatic Cu	Elevated	Normal	Normal	Low
65Cu test	Abnormal	Abnormal	Abnormal	Abnormal
				Normalize after i.v. Cu
CuEXC	Normal to high	Normal	Normal	Likely low
REC	High	High	High	Likely normal

No treatment of HB has been described. The patient has tolerated zinc therapy for 26 years without symptoms of copper deficiency and lived longer than any other patient with HB. On presentation in 1996, 24H U-Cu was moderately elevated. On this background, we decided to slowly taper the chelation therapy under close monitoring of symptoms and copper status.

## Discussion

We present a 53-year-old woman with a phenotypically milder form of HB who was mistaken for WD for 25 years.

The suspicion of milder HB was raised when genetic analysis did not support the diagnosis of WD (*ATP7B*) or aceruloplasminemia (*CP gene)*. Whole genome sequencing detected two variants of uncertain significance in the *SLC33A1* gene indicative of HB. These variants were predicted to cause dysfunction of AT-1 potentially affecting the synthesis of a number of proteins, including the absence of ceruloplasmin.

The previous pediatric cases of HB also showed low ceruloplasmin, low P-Copper, and normal hepatic copper content (1, 3). Since nearly all copper is complexed within ceruloplasmin, low S-Cu is expected in HB because ceruloplasmin is low (2). Since ATP7B function is normal in HB, biliary excretion is maintained which explains normal hepatic copper (2). During the ^65^Cu test, ^65^Cu first disappears from plasma and then reappears after 72 h due to the formation of ceruloplasmin. The second peak is absent in WD but also in other conditions with low ceruloplasmin such as aceruloplasminemia and HB. Exchangeable or “free” copper, CuEXC, was normal, while the ratio of free *vs*. total copper, REC, was elevated because total copper was low (15). That would also be the case in WD.

In contrast to our patient, normal 24H U-Cu was reported in pediatric cases, but U-Cu quantification is uncertain in children (1, 3).

Disturbed copper metabolism may not be of pathophysiologic importance in HB. The AT-1 transporter is expressed in many tissues including brain and spinal cord and affects the function of a number of proteins (17). This may explain the neuropsychiatric symptoms in our patient who showed similarities with previously reported childhood cases (e.g., developmental delay, hearing loss, and ataxia). This case does not fully meet the previous reports of HB as she has no congenital cataract and survived into adulthood. MRI findings were inconclusive. Without pediatric MRI, it is impossible to determine whether the global atrophy seen in this case was acquired in adulthood; however, it may have been congenital in which case it would be described as hypoplasia. The wide subarachnoid spacing is a result of either hypoplasia or atrophy. Hypomyelination is primarily a term used in pediatrics and may refer to late myelination. While this subject did not show congenital hypomyelination nor acquired demyelination, late myelination would not be expected to be visible in the adult brain.

The previously reported homozygous and compound homozygous variants leading to exon skipping and/or premature termination of translation or alternatively mislocalization of the SLC33A1 protein variant might be very destructive for the protein product (1, 18). While these were associated with neurologic disease in childhood, a publication reported that missense mutation in SLC33A1 (p. S113R) leads to dominantly inherited spastic paraplegia (19). The presently identified variants: an in-frame deletion of a single amino acid combined with and a missense variant in the *SLC33A1* gene, might be less destructive for the protein and explain this milder clinical presentation of the HB. Thus, the present case serves to expand the phenotypic spectrum for the sparse number of patients with *SLC33A1* variants (1, 3, 19).

Because of the original WD diagnosis, the patient received zinc therapy for many years. Whether this affected her disease course is unclear, but she developed no signs of copper deficiency. Her life-threatening deterioration in 2017 was likely caused by an overdose of antipsychotics.

This case serves to expand the clinical picture of HB and highlights how adult HB may easily be mistaken for WD as well as the need for more specific diagnostic tools. Furthermore, this case serves as a reminder that patients may fulfill the Leipzig criteria without having WD. Thus, these patients may be found in the populations treated for WD. The newly described ^64^Cu PET/CT method may be useful for secure diagnosis but remains experimental and was not performed on this patient (20).

## Data availability statement

The datasets presented in this article are not readily available because of ethical and privacy restrictions. Requests to access the datasets should be directed to the corresponding author.

## Ethics statement

Ethical review and approval was not required for the study on human participants in accordance with the local legislation and institutional requirements. The patients/participants provided their written informed consent to participate in this study. Written informed consent was obtained from the patient for the publication of any potentially identifiable images or data included in this article.

## Author contributions

FK wrote the manuscript and acquired patient data. JE and LB aquired genetic data. MB analyzed patient MRI. EH analyzed patient neurology. DM, JE, LB, MB, EH, and TD assisted in writing the manuscript. HV, PO, and TD helped form the project and gave inputs to the manuscript. All authors contributed to the article and approved the submitted version.

## Conflict of interest

The authors declare that the research was conducted in the absence of any commercial or financial relationships that could be construed as a potential conflict of interest.

## Publisher's note

All claims expressed in this article are solely those of the authors and do not necessarily represent those of their affiliated organizations, or those of the publisher, the editors and the reviewers. Any product that may be evaluated in this article, or claim that may be made by its manufacturer, is not guaranteed or endorsed by the publisher.
